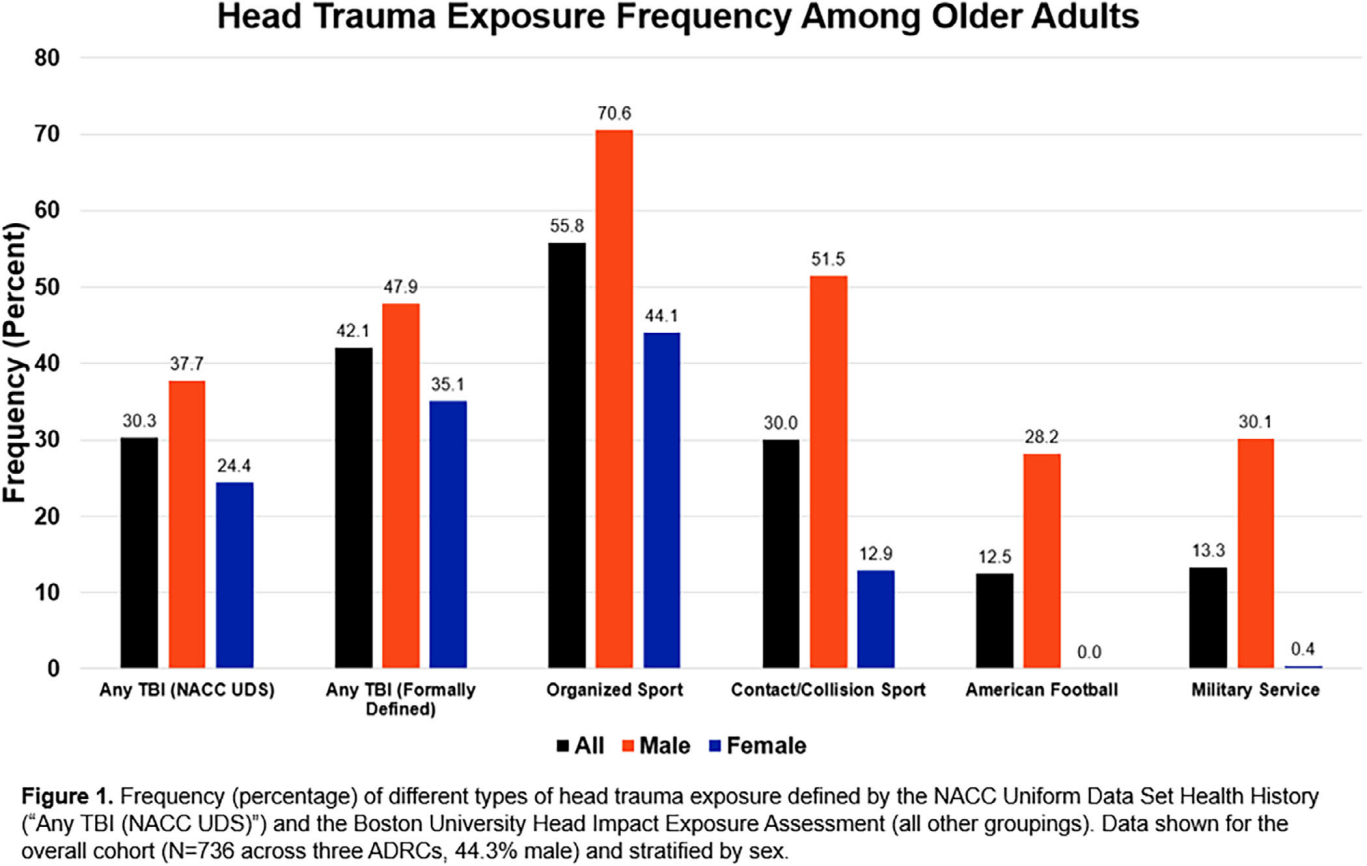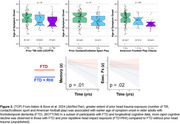# Unrecognized Repetitive Head Impact Exposure Among Older Adults Across Three Alzheimer's Disease Research Centers

**DOI:** 10.1002/alz70860_096743

**Published:** 2025-12-23

**Authors:** Breton M. Asken, Jessica Bove, Jamie Blackband, Glenn E. Smith, Joel H Kramer, Gil D. Rabinovici, Anna Aaronson, Thor D. Stein, Ann C. McKee, Jesse Mez, Michael L Alosco

**Affiliations:** ^1^ Department of Clinical and Health Psychology (B.M.A.), University of Florida, Gainesville, FL, USA; ^2^ University of Florida, Gainesville, FL, USA; ^3^ 1Florida Alzheimer's Disease Research Center, Miami, FL, USA; ^4^ Memory and Aging Center, UCSF Weill Institute for Neurosciences, University of California, San Francisco, San Francisco, CA, USA; ^5^ UCSF Alzheimer's Disease Research Center, San Francisco, CA, USA; ^6^ Memory and Aging Center, Weill Institute for Neurosciences, University of California San Francisco, San Francisco, CA, USA; ^7^ Boston University CTE and Alzheimer's Disease Research Center, Boston, MA, USA; ^8^ Boston University Alzheimer's Disease Research Center, Boston, MA, USA; ^9^ Boston University Chronic Traumatic Encephalopathy Center, Boston University Chobanian & Avedisian School of Medicine, Boston, MA, USA; ^10^ Boston University Chronic Traumatic Encephalopathy Center, Boston, MA, USA; ^11^ Alzheimer's Disease Research Center, Boston University Chobanian & Avedisian School of Medicine, Boston, MA, USA, Boston, MA, USA; ^12^ Boston University Chobanian & Avedisian School of Medicine, Boston, MA, USA

## Abstract

**Background:**

Repetitive head impact exposure (RHI) is the only known risk factor for chronic traumatic encephalopathy (CTE), a distinct neurodegenerative tauopathy. Most aging studies ask participants about prior traumatic brain injury (TBI), but not common sources of RHI. Understanding the public health footprint of RHI requires targeted questioning of lifetime exposure. To set the stage for our Featured Research Session on CTE, we report RHI prevalence using gold standard methods across independent aging and dementia cohorts.

**Method:**

The Boston University Repetitive Head Impact Exposure Assessment (BU‐RHIEA) was administered across three cohorts: BU Alzheimer's Disease Research Center (ADRC), UCSF ADRC, and 1Florida ADRC. Participants recruited explicitly with concerns about cognitive effects of prior head trauma were excluded. The BU‐RHIEA queries participation in contact/collision sports (e.g., American football, soccer, ice hockey, boxing) and military service as well as prior TBI. We report RHI and TBI prevalence estimates from the BU‐RHIEA relative to estimates from the National Alzheimer's Coordinating Center Uniform Data Set (NACC UDS). In a subset of participants with frontotemporal dementia, we evaluated the clinical relevance of prior RHI through associations with age of symptom onset and rate of disease progression.

**Result:**

Data come from 736 participants (age 70.4±9.6, 44.3% male, 43.1% cognitively impaired). Based on NACC UDS, 223 (30.2%) reported any prior TBI (Figure 1). When providing a formal TBI definition, frequency increased to 300 (40.8%). Sport and military history are not known through NACC UDS. Based on the BU‐RHIEA, 221 (30.0%) participants reported any prior contact/collision sport play. Among males, 92 (28.2%) previously played American football and 98 (30.1%) served in the military. In participants with frontotemporal dementia, prior RHI related to younger symptom onset and more rapid cognitive decline (Figure 2).

**Conclusion:**

There is a high risk of underestimating the scope of RHI influence on brain aging. Among older adults, history of TBI and RHI from popular contact/collision sports is exceedingly common but unrecognized. RHI may contribute to the wide variability observed in the onset and progression of neurodegenerative conditions. Studying head trauma exposure relevant for later‐life brain health, such as sport‐ and military‐related RHI, requires dedicated questioning.